# Supplementary material for: Are carbon emissions trading and green financial instruments synergistic? -Comprehensive quantitative research based on content analysis
Source: PLoS One. 2024 Mar 7;19(3):e0298601. doi: 10.1371/journal.pone.0298601 (PMC10919601; doi:10.1371/journal.pone.0298601)
Supplement: S1 Data — (ZIP) [file pone.0298601.s001.zip › Data/地级市Z_F工作报告.zip!/╣π╬≈/╬α╓▌╩╨/└┤╫╘╣1⁄2╓┌║┼í╛┬φ┐╦╩2╛▌═°í┐_2012.docx]

各位代表：

　　现在，我代表市人民政府向大会作政府工作报告，请予审议，并请市政协委员和其他列席会议的同志提出意见。

　　2011年工作回顾

　　过去的一年，面对严峻复杂的国内外形势，我们在自治区党委、政府和市委的正确领导下，深入贯彻落实科学发展观，抢抓发展机遇，积极应对挑战，奋力开拓进取，全面推进富民强梧新跨越，圆满完成了全年各项目标任务，实现了“十二五”良好开局。

　　──地区生产总值持续高速增长。预计完成地区生产总值690亿元，同比增长15%，高于全区平均水平2个百分点，连续三年保持15%以上的增速。

　　──规模以上工业总量实现两年翻番。全部工业总产值和规模以上工业总产值均突破千亿元大关，预计规模以上工业总产值1036亿元，比2009年翻1.1番，同比增长47.5%，增速排全区第3位；规模以上工业增加值358亿元，比2009年翻1.2番，同比增长26%，增速排全区第3位。

　　──财政收入实现三年翻番。预计完成财政收入76.1亿元，比2008年翻1.7番，同比增长35.6%，增速排全区第2位。其中一般预算收入52亿元，比2009年翻1.1番，同比增长36%。岑溪市和藤县财政收入均突破10亿元大关。

　　──固定资产投资实现三年翻番。预计完成全社会固定资产投资632亿元，比2008年翻1.7番，同比增长34.9%，投资完成进度连续三年排全区第1位。

　　──金融机构贷款余额实现三年翻番。预计年末金融机构各项贷款余额396亿元，比2008年翻1.1番，比年初增长22.9%，增速排全区第2位。

　　──招商引资到位资金实现三年翻番。全市招商引资实际到位资金355.6亿元，比2008年翻1.3番，同比增长25.7%。

　　──园区产值实现一年翻番。新增产值超百亿元的园区3个，园区工业总产值达705亿元，比上年翻1.3番，占全市工业总量的58%。

　　──城乡居民收入不断提高。预计城镇居民人均可支配收入18239元，同比增长11%；农民人均纯收入5610元，同比增长15%。财政用于民生项目的支出达59亿元，占财政总支出的49.6%。

　　──城市影响力不断提升。成为广西首个“国家森林城市”、广西唯一的“国家城市矿产”示范基地，第4次荣获“全国双拥模范城”称号，获得“全国文明城市”提名。

　　我们在富民强梧新跨越的道路上迈出了更加坚实的一步！

　　一年来，我们主要做了以下工作：

　　一、大力推进新型工业化。着力打造重点产业集群，年产30万吨再生铜冶炼工程一期、年产3万吨铜棒铜管、永达100万吨不锈钢制品技改一期、华南再生棉一期等项目竣工投产，金海100万吨、东鹏120万吨镍铬合金制品、理文10万吨差别化化学纤维、鸿图2万吨精密压铸件等项目开工建设。全力扶持优势企业做大做强，规模以上工业企业达430家，比上年增加30家，重点扶持的102家企业工业总产值、主营业务收入和实现利润同比分别增长120%、99%和60%。加大企业技改和新产品开发力度，提高企业自主创新能力，国家级高新技术企业从9家发展到14家，有8家企业列入广西创新型试点企业，15家企业列入广西制造业信息化工程示范企业，2家企业被认定为广西优秀民营创新型企业。“五园八区”建设加快推进，梧州电子信息产业园建设全面启动。

　　二、大力推进农业现代化。粮食总产量达80.87万吨，增产4867吨。加快建设西江经济带特色农业示范区，砂糖桔、六堡茶和中草药种植面积达70万亩，规模养殖场达4028个。加快农业产业化进程，新增1家国家级、3家自治区级、14家市级农业产业化重点龙头企业。完成集体林权主体改革，林下经济收益达32亿元。统筹推进新农村建设和城乡风貌改造，全市新农村建设示范点扩大到30个。加大民生水利投入，城市防洪、水库除险加固、农村安全饮水等项目共完成投资6.76亿元，同比增长48.9%。实施土地整理项目6个，完成投资1.2亿元。

　　三、大力推进现代服务业发展。加快商贸流通项目建设，市烟草物流配送中心投入使用，红岭商贸物流园、中恒国际医药物流城等项目顺利推进，全年安排商贸流通项目64个，完成投资29.38亿元。推进旅游业和文化产业的融合发展，市博物馆新馆建成开馆，改造了珠山公园，新建了中恒双钱龟苓膏、茂圣六堡茶工业旅游点。加快发展金融服务业，北部湾银行、招商证券进驻梧州。努力拓展城乡消费空间，预计完成社会消费品零售总额222.5亿元，增长16%。

　　四、大力推进基础设施建设。南广铁路梧州段、长洲水利枢纽三线四线船闸、旺村水利枢纽一期工程等重大项目进展顺利，梧州至贵港、岑溪至水汶高速公路加快建设，梧州至柳州、市环城高速公路、国家西气东输二线工程梧州段开工建设，梧州机场迁建工程、柳州至肇庆铁路等项目前期工作加快推进。李家庄码头三期、紫金村综合码头、大利口码头等项目开工建设。全年港口吞吐量2071万吨，集装箱吞吐量27.8万标箱，同比分别增长29%和38%，西江黄金水道建设取得重大进展。

　　五、大力推进新型城镇化。着力打造生态宜居城市，积极开展“国家园林城市”创建活动。“三冲”地质灾害综合整治工程加快推进，搬迁4404户，安置20600人，累计投入8.11亿元。加快红岭新区建设，路网工程22条开工、13条贯通，玫瑰湖整治一期工程竣工；加快南岸开发，西江三桥、苍海等项目顺利推进，西江四桥、南广铁路梧州站站前广场开工建设，新增城区面积6平方公里。深入开展城乡环境综合整治，岑溪市“一河两岸”建设加快，藤县藤州大道拓宽工程完工，蒙山县桂北民居建筑风格初步形成。全市城镇化率达到47%，高于全区平均水平5个百分点。

　　六、大力推进开放合作。全面实施“东向”战略，桂东承接产业转移示范区建设取得积极进展，签署了《肇庆-梧州战略合作框架协议》，推动粤桂合作特别试验区上升为两省（区）发展战略。成功承办广西纪念辛亥革命100周年学术研讨会，举办了第八届梧州国际宝石节、第四届西江经济发展论坛和第四届广西（梧州）春茶节暨六堡茶博览交易会。全年引进外来投资项目326个，投资总额357.1亿元；实现外贸进出口总额7.96亿美元，增长24%。

　　七、大力推进生态文明建设。实施“绿满八桂”工程，大规模造林绿化，完成造林23.6万亩，获得“广西绿化模范城市”称号。完成自治区下达的节能减排年度目标任务，削减化学需氧量1654吨，削减氨氮排放量281吨，江河水质达标率保持100%，空气质量一级天数位列全国各城市第3位。启动城镇垃圾分类收集和分选处理系统建设，积极推广城市生活小区垃圾分类回收工作。岑溪市获“全国生态文明先进县（市）”称号。

　　八、大力推进民生建设。实施教育惠民工程，投入资金4.27亿元，其中1.17亿元用于资助13万名贫困学生上学。职教攻坚顺利通过自治区评估验收，获“全区职教攻坚先进单位”称号。实施卫生保障惠民工程，重点推进的11项基本公共卫生服务项目惠及全体城乡居民，在全区率先成立市级新型农村合作医疗管理中心，实现医疗费用即时结算报销。实施社会保障惠民工程，城乡居民养老保险覆盖面达60%。实施“安居”工程，开工建设保障性住房11230套，完成11700户农村危房改造任务，为全市65万农户实行住房政策性保险。实施“乐业”工程，发展微型企业2362家，新增就业10.46万人，城镇登记失业率低于自治区控制指标。实施文化惠民工程，完成中山纪念堂、李济深故居改造工程，建设农村公共服务中心84个、城市社区公共服务中心10个、农家书屋421个。加强物价调控，对猪肉、大米、食用油等生活必需品实行限价销售，给城乡困难群众发放临时价格补贴，全年居民消费价格总水平涨幅为5.4%，低于全区平均水平。“千村万户”体育活动覆盖城乡，成功申办广西第十三届运动会。创新人口计生社会管理机制，人口自然增长率控制在自治区下达的责任指标以内。

　　九、大力推进平安梧州建设。加强和创新社会管理，城市地质灾害综合整治、肇事肇祸精神病人管理、刑释解教人员安置帮教等工作得到了中央和自治区的高度评价。建立健全“四个一”矛盾纠纷排查调处体系，全市矛盾纠纷调处成功率达到95%。组织开展“清网行动”、“打四黑除四害”等专项行动，刑事案件破案率稳步提高。推进复合型警务机制改革，在市区设立6个警务工作站，市区刑事案件发案率明显下降。深入开展安全隐患排查和专项整治，安全生产形势总体平稳。加大食品药品监管力度，保障人民群众饮食和用药安全。综治工作进入全区一等奖行列，社会公众安全感单项测评排全区第4位，连续六年被自治区授予“广西平安市”称号。

　　一年来，政府自身建设和民主法制建设不断加强。自觉接受人大及其常委会的法律监督、工作监督，主动接受政协的民主监督，共办理人大代表建议100件、政协委员提案260件，按时办结率100%。深入开展行政效能建设，组织对30个窗口部门及100名科长开展大评议活动，对37个相关部门单位进行公众满意度测评。市、县、乡政府换届工作圆满完成。强化经济责任及政府投资项目审计监督，反腐倡廉工作取得新成效。

　　国防教育和国防后备力量建设不断加强，驻梧部队在支持地方建设、处置突发事件等方面作出了重要贡献。中直、区直驻梧各单位为经济社会发展提供了大力支持。民族、宗教、外事、侨务、对台、人防、消防、口岸、统计、供销、地震、扶贫、农机、社会科学、市志、档案、妇女儿童、老龄、残疾人等工作取得新进步，工会、共青团、妇联等群团组织重要作用得到充分发挥。

　　各位代表，“十二五”开局之年，我们经受住了各种严峻考验，巩固和扩大了经济社会平稳较快发展的良好势头，成绩来之不易。这是自治区党委、政府和市委坚强领导的结果，是市人大、市政协和社会各界监督支持的结果，是全市人民同心协力、克难奋进的结果。在此，我代表市人民政府，向全市人民，向人大代表、政协委员，向所有关心、支持、参与梧州建设的同志们、朋友们表示崇高的敬意和衷心的感谢！

　　在肯定成绩的同时，我们也清醒地认识到，我市经济社会发展中还存在不少困难和问题。主要是：电力供需矛盾突出，项目融资困难，用地指标紧张，保持投资较快增长难度加大；现代物流业发展相对滞后于工业产业发展的问题开始显现；政府公共服务能力和水平有待进一步提高等。对此，我们一定高度重视，千方百计加以克服和解决。

　　2012年主要目标任务

　　2012年，是实施“十二五”规划承上启下的重要一年，也是我市推进富民强梧科学发展三年计划的关键之年。当前，世界经济复苏的不稳定性不确定性上升，经济形势仍将异常严峻复杂。国内经济发展中不平衡、不协调、不可持续的矛盾和问题仍然突出，经济增长下行压力和物价上涨压力并存，转方式调结构的任务十分迫切。同时，我们也应看到，随着国家实施新一轮西部大开发，启动新10年扶贫开发，支持东部产业转移，以及自治区深入实施“两区一带”发展战略，我市将迎来多种优惠政策叠加的发展机遇期，新型城镇化的加速期，扶贫攻坚的关键期。我们既要充满信心，又要保持清醒，充分利用好各种有利条件，锐意进取，奋发有为，不断开创经济社会发展的崭新局面。

　　根据市第十二次党代会和全市经济工作会议的部署，2012年政府工作的总体要求是：以邓小平理论和“三个代表”重要思想为指导，深入贯彻落实科学发展观，围绕建设“两城市一基地”的战略目标，以科学发展为主题，以加快转变经济发展方式为主线，坚持稳中求进、好中求快，走好经济发展“三步棋”，唱响城市建设“三部曲”，以“三年一工程”为抓手，千方百计稳增长、强产业、固基础、扩城镇、惠民生、促和谐，保持和扩大经济社会发展的良好势头，加快推进富民强梧新跨越，以优异成绩迎接党的十八大胜利召开。

　　全年经济社会发展的主要预期目标是：地区生产总值增长13%，力争15%；全社会固定资产投资增长25%，力争30%；财政收入增长20%，力争26%；规模以上工业增加值增长22%，社会消费品零售总额增长17%，城镇居民人均可支配收入增长12%，农民人均纯收入增长13%，城镇登记失业率控制在4.5%以内，完成自治区下达的节能减排任务，人口自然增长率控制在10％。以内，居民消费价格总水平涨幅控制在4%左右。

　　做好今年的政府工作，必须主动适应形势的新变化，立足科学发展的新起点，切实增强机遇意识、忧患意识、危机意识和责任意识，正确处理稳与进、好与快的关系，以时不我待、只争朝夕的精神，在解放思想中拓宽视野，在坚定信心中激发动力，在奋力赶超中争先进位。着力在推进转方式调结构上有新进展，在深化改革扩大开放上有新突破，在统筹经济社会发展上有新成效，在保障和改善民生上有新作为，努力实现经济总量更大、发展质量更好、群众收入更多、社会保障更全、文明程度更高、人民幸福感更强。

　　2012年主要工作安排

　　实现今年的目标任务，必须重点抓好以下9项工作：

　　一、强产业调结构

　　加快培育千亿元产业。加快发展再生资源产业，重点推进30万吨再生铜、30万吨再生铝、1000万台蓄电池和2万吨精密压铸件等项目，力争年内实现产值200亿元。加快发展不锈钢制品产业，重点推进金海100万吨、东鹏120万吨镍铬合金制品、永达100万吨不锈钢制品技改二期等项目，力争年内实现产值300亿元。加快发展以掌上多媒体为核心的电子信息产业，推动五洲（株）电路板、鑫华通手机等重大项目落户，支持深圳手机协会进驻梧州，加快海德威电子信息基地、创维集团电子信息研发基地等项目扩容升级，力争电子信息产业实现产值100亿元以上。力争到2015年再生资源、不锈钢制品、电子信息三大产业产值均达到千亿元。

　　加快发展百亿元产业。加快陶瓷产业发展，重点推进藤县新舵陶瓷、岑溪新动力陶瓷等项目建设，力争年内实现产值115亿元，到2015年产值超200亿元。以中恒制药集团、神冠蛋白肠衣公司为龙头，加快发展制药、食品产业集群，力争到2015年两大产业产值分别超200亿元。加快推进藤县年产10万吨金红石钛白粉和年产10万吨酸钛联产异地技改、万秀区3万吨高等级交通标志涂料生产、松脂深加工等项目，力争化工产业实现产值125亿元。加快推进港口集装箱生产基地、长洲区三一重工工程机械整机制造项目建设，力争机械制造产业实现产值125亿元。以石材、管材、建筑装饰材料为重点，做大建材产业，力争年内实现产值190亿元。

　　加快实施“抓大壮小扶微”工程。加大扶持大企业大集团的力度，力争年产值30亿元以上的企业达9家、10至30亿元的企业17家、5至10亿元的企业24家，形成梯次强企方阵。积极引导、推动主营业务收入2000万元以下的企业通过增加投入、盘活存量、兼并重组等方式做大规模，力争新增规模以上企业50家，总数达到480家。全面落实微型企业扶持政策，大力发展现代物流、服务外包、电子商务、民生服务、农产品加工等微型企业，新增微型企业2000家。

　　加快发展现代服务业。加快构建西江经济区域商贸物流中心，安排商贸流通项目50个，总投资222.24亿元，年度完成投资60.28亿元。重点推进红岭商贸物流园、中恒国际医药物流城、毅德现代商贸城、电子产业物流项目、再生资源集散交易市场等项目。加快推进沃尔玛、国美电器、梅西百货等品牌商贸项目，建设一批休闲商务区、特色商业街。深入推进“万村千乡市场工程”和农贸市场建设改造。加快金融服务业发展，积极引进各类金融机构在我市设立分支机构，提高金融服务质量和效率。大力发展信息服务业，开工建设中国联通东盟梧州分中心、梧州移动无线城市和电信光网城市等信息通道，拓展电子政务、电子商务、数字城市等信息平台，打造西江信息枢纽港。加快建设旅游目的地和游客集散地，按照“一核四组团”和“10+2”旅游景区发展格局，加快推进华际亚太影视城、西江游艇基地、苍梧六堡茶产业化展示区等项目建设，力争旅游项目完成投资25亿元以上。

　　加快发展文化事业和文化产业。坚持先进文化的前进方向，弘扬“团结和谐、爱国奉献、开放包容、创新争先”的广西精神。加强骑楼城等文化遗产的保护，挖掘西江文化内涵，推进西江文化博物馆、城市地质灾害博物馆、文化大厦、新图书馆建设。促进文艺精品创作，承办广西第一届粤剧节。推进文化体制改革和创新，开发文化旅游、文化创意、文化会展、演艺娱乐、广播影视等文化产业。力争文化产业增加值占GDP比重达到3.3%。

　　二、稳农业保增收

　　着力发展优势特色农业。稳定粮食生产，确保粮食种植面积达237万亩，粮食总产量80万吨以上。继续实施优果工程，力争水果产量达40万吨，增长10%以上。稳定“菜篮子”基地1万亩，发展品牌蔬菜6000亩，发展六堡茶标准化茶园2000亩以上。建设水产畜牧业示范园区，加快引进中粮集团年产50万头肉猪及配套饲料厂项目，推进生猪、三黄鸡、肉兔、养蜂等特色养殖业标准化、规模化，力争到2015年畜牧水产业占农业总产值比重达到50%以上。大力发展林业产业集群，培育1000万亩原料林基地，加快推进理文10万吨差别化化学纤维项目，实现林业总产值180亿元。积极培育农民专业合作组织，新发展各类专业合作社50家以上。

　　着力改善农村生产生活条件。实施民生水利工程，筹措资金8.44亿元，新增解决20万农村人口饮水安全问题，完成渠道防渗配套550公里，新增恢复灌溉面积5.58万亩，改善灌溉面积25.5万亩，新建加固堤防38公里，实施79座病险水库除险加固。实施新一轮新农村建设，重点抓好20个示范点的水、电、路、广播电视、信息网和公共服务中心建设。加强农村环境综合整治，逐步推行农村生活垃圾“村收-镇运-县处理”机制。加快推进城乡电网升级改造和城乡水务一体化。

　　着力拓宽农民增收渠道。深入推进“万元增收工程”，建设20万亩吨粮“万元田”、20个“一村一品”高效特色农业和休闲农业“万元村屯”、60个50亩以上集中连片的特色种植“万元片区”，培育60个年纯收入10万元以上的致富示范户。鼓励和帮扶外出务工农民回乡创办加工、商贸流通、储运等“农家店”实体，大力发展林下经济、庭院特色养殖和乡村旅游等，增加农民经营性收入。加强农业职业教育，促进农村劳动力转移，增加农民工资性收入。落实各项惠农强农政策，增加农民政策性收入。探索建立农村土地经营权、林权流转和抵押机制，增加农民财产性收入。

　　着力实施新一轮扶贫开发。开展以产业扶贫、基础设施扶贫、信贷扶贫、培训扶贫为重点的“整村推进”扶贫攻坚工作，巩固和扩大第一批90个贫困村的扶贫开发成果。做好库区移民安置工作，实施移民增收项目，力争增收项目和资金增长30%以上。水库移民后期扶持5万人，补助资金2800万元。整合各类涉农资金，动员社会各方面力量，努力形成专业扶贫、行业扶贫、社会扶贫三位一体的大扶贫格局。

　　三、增投资上项目

　　千方百计扩大投资。继续开展“项目攻坚年”活动，计划安排重点建设项目861个，总投资3265.4亿元，年度计划完成投资688.4亿元。其中，续建项目402个，年度计划投资353亿元；新开工项目378个，年度计划投资335.4亿元；重点前期项目81个，总投资1009.6亿元。总投资亿元以上项目381个，年度计划投资亿元以上项目159个。加大对保障性安居工程、教育、卫生、水利等民生项目的投入，大力支持“三农”、产业园区、商贸物流、社会事业等领域的基础设施建设，力争完成全社会固定资产投资820亿元，增长30%。

　　强化要素保障。完善审批部门联席会议、项目业主培训、中介咨询机构规范管理及项目代办等制度。完善“五个一”工作机制，全面推行“一线管理法”、“协同管理法”，强化重大项目全程跟踪服务。完善气电运协调机制，强化项目用地保障，推进集约节约用地。完善“政银企”协调机制，加大信贷投放力度。出台企业上市扶持政策，推进奥奇丽、日成林化、梧州松脂、金茂钛白等企业上市。加强投融资平台规范管理，开辟多元化筹资渠道。

　　加大招商引资力度。继续开展“园区建设（招商）年”活动，组建十大产业的招商工作队，以珠三角为重点区域，围绕优势产业和特色产业开展产业集群招商。创新招商方式，突出抓好专题招商、以商引商、节会招商、产业链招商，集中在战略性新兴产业、现代服务业、城市开发建设等领域招大引强。力争全年招商引资实际到位资金增长30%以上。

　　四、兴交通固基础

　　加快建设“一环七射三连线”高速公路网。投资26.5亿元，全面加快梧州至贵港、梧州至柳州、岑溪至水汶高速公路和市环城高速公路建设，启动梧州至省界接广佛肇高速公路的前期工作，推进梧州至信都高速公路前期工作，力争荔浦至铁山港高速公路梧州段开工建设。

　　加快建设“丰字型”铁路网。投资10亿元，加快南广铁路梧州段、岑罗铁路等续建项目建设，推动梧州港赤水圩作业区码头进港铁路专线实质性开工，加快推进柳州至肇庆铁路梧州段、柳州至韶关铁路梧州段等项目前期工作。

　　加快建设西江黄金水道。投资13.5亿元，重点推进李家庄三期、紫金村、大利口和中储粮等坝下港口码头建设，坝下新建千吨级以上泊位17个，其中3000吨级泊位6个、2000吨级泊位5个、1000吨级泊位6个；加快建设梧州港赤水圩作业区二期工程，积极推进藤县三坡码头、东胜作业区等项目前期工作。加快长洲水利枢纽三线四线船闸工程建设，建成旺村水利枢纽。积极推动贵港至梧州、梧州至肇庆3000吨级航道整治等项目前期工作。

　　加快建设梧州航空枢纽港。新增梧州至柳州、福州、深圳航线。完成梧州机场迁建工程前期工作，实现年内开工建设，努力建设辐射桂东地区的航空运输枢纽。

　　加快改造市域县际路网。推进龙圩至潭东二级公路改一级公路、苍郁高速公路大坡互通立交、梧州至倒水段公路提级改造等项目建设。加快推进农村公路村村“通畅”工程，新增50个行政村通硬化路，通村公路硬化率达73%以上。启动蒙山县城至夏宜乡二级公路建设，推动岑溪207国道过境一级公路、藤县赤水沿江一级公路、三龙至津北沿江二级公路等项目前期工作。

　　五、扩城镇提品位

　　抓好中心城市建设。全面建设红岭新区，重点推进中央商务区、新加坡印象、玫瑰湖公园等项目。全面启动南岸片区开发建设，重点加快苍海、高旺片路网、南广铁路梧州站站前广场、西江三桥、西江四桥等项目建设，力争上半年苍海项目实现“成湖成景”。启动城市取水点上移工程，力争年内开工建设北山、富民水厂迁建工程，完成龙华水厂项目前期工作。加快国家西气东输二线梧州段项目建设，全面推进天然气入梧工程，推广天然气综合利用。推进旧城改造工程，加快石鼓冲地质灾害综合整治。规划建设应急避难场所及配套设施。

　　抓好县城和重点镇建设。加快构建城市带动城镇群、城镇群带动广大农村协调发展的新格局。推进苍梧与中心城区同城一体化，加快岑溪、藤县和蒙山城市化步伐，支持苍梧县龙湖世纪城二期、岑溪“一河两岸”中心区市政基础设施、藤县碧水湾小区、蒙山华虹滨江新城等重点项目建设，促进产业布局、交通组织、公共设施、生态环境与人口聚居相协调。支持石桥、太平、归义、黄村等重点小城镇完善功能配套，增强人口和要素吸纳能力，发挥小城镇在统筹城乡发展中的传递作用。

　　抓好城市综合管理。坚持规划、建设与管理并重，提高城市现代化水平，优化城市组团功能定位和建设风格。深化“国家森林城市”建设，构建城市森林景观带，促进城市与山系、江河、绿地和谐共生，创建“全国文明城市”、“国家园林城市”。加大对违法用地和非法建设的打击力度，持续开展城市环境综合整治，确保城乡清洁工程全域覆盖、保持常态。深入开展城市生活垃圾分拣工作，进一步完善城市生活垃圾无害化处理机制，建成压缩式生活垃圾中转站6座。探索推进城市管理权限下放，强化城市数字化和城区网格化管理。

　　六、抓改革促开放

　　加快建设产业承接平台。深入实施“东向”战略，全方位加强与珠三角地区的产业、交通、项目等对接合作，全面落实梧肇战略合作框架协议，推动粤桂合作特别试验区建设，同时加强与广东佛山、云浮等城市的战略合作。抓住自治区深入实施桂东承接产业转移示范区发展规划的机遇，全力争取政策、项目和资金支持，加快承接产业转移示范基地建设。推进“五园八区”提质扩容，完成基础设施投资24亿元以上，提升园区基础设施建设的整体水平和产业配套能力。力争园区产值超千亿元，占全市工业总量的比重达到65%以上。

　　扩大开放合作。充分利用中国-东盟自由贸易区平台，进一步拓展与东盟合作交流。着力提升梧州国际宝石节影响力，办好中国茶业学会暨六堡茶博览交易会。加快国家加工贸易梯度转移重点承接地建设，重点做好高新技术产业园区、出口加工区、梧州港赤水圩作业区码头口岸监管区申报工作。力争实现外贸进出口总额9.6亿美元，增长20%。

　　深化重点领域改革。深化财政、金融管理改革，加强公共预算体系建设，推动地方金融机构改革纵深发展。推进农村金融改革试点和社会信用体系建设。稳步推进资源性产品价格和环保收费改革。推进集体林权配套改革，完善农村土地流转机制。

　　七、重环保优生态

　　积极发展循环经济。加快“国家城市矿产”示范基地建设，推进工业园区和重点企业的循环化改造。积极构建再生资源回收体系，加快60万台家电拆解项目建设，规划建设再生资源回收站（点），争取成为国家第三批再生资源回收体系建设试点城市。探索新型资源开发利用模式，积极发展光伏电子、建材、有色金属、生物制药等循环经济产业。大力推广以沼气为纽带的农业循环经济发展模式，重点打造养殖-沼气-种植、农林产品加工及资源化利用等循环产业链。大力倡导绿色低碳的生活方式，开展节能环保行动，发展绿色生态的现代服务业。

　　强力推进节能减排。严格执行高耗能产业准入条件，加强节能减排新技术、新产品、新工艺、新材料的推广应用，申报国家级可再生能源建筑应用示范城市。加快建设市第二污水处理厂二期工程、第三污水处理厂和小城镇污水处理厂。加大西江、桂江等重要水源地保护力度，抓好主要污染物减排工作，确保空气质量优良率、江河水质达标率均保持100%。

　　加强生态环境建设。深入实施“绿满八桂”工程，推进城市干道绿化美化、街道小区与庭院绿化、城市通道与江河两岸景观绿化、城市公园与公共休闲绿地建设，重点提升西江、桂江沿岸和南梧高速公路两旁绿化景观。积极开展农村环境连片整治示范县试点工作。广泛开展国家生态文明示范城市、国家环保模范城市、生态村镇、绿色学校和绿色社区创建活动。

　　八、办实事惠民生

　　把保障和改善民生摆在更加突出的位置，确保民生投入增长高于财政经常性收入增长，确保新增财力更多用于社会事业和民生改善。今年筹措资金30亿元以上，集中力量为人民群众办好12件实事：

　　1．教育惠民工程。筹措资金1.68亿元，改造维修农村义务教育阶段学校校舍和建设农村幼儿园，对农村义务教育阶段经济困难生、寄宿生和普通高中贫困学生进行资助，实施农村义务教育学生营养改善计划。

　　2．卫生惠民工程。筹措资金5.59亿元，提高新型农村合作医疗筹资标准，建设标准化的城市社区卫生服务中心。深入实施防治艾滋病攻坚工程、母婴健康“一免二补”工程和地中海贫血防治计划，继续实施农村改厕项目。

　　3．社保惠民工程。筹措资金1.26亿元，实现城乡居民社会养老保险制度全覆盖，对参保人员参保缴费给予适当补贴；对60周岁以上、符合条件的城乡居民按政策规定及时足额发放基础养老金；新建113个五保村；实施残疾人“阳光家园计划”项目，为5000名符合条件的智力、精神和重度残疾人提供居家托养和日间照料服务。

　　4．文化惠民工程。筹措资金3568.8万元，建设35个村级公共服务中心和172个农家书屋，实施农村电影公益放映、广播电视村村通和农村地面数字电视覆盖工程。

　　5．安居惠民工程。筹措资金18亿元，开工建设保障性住房1.117万套、总面积69万平方米，重点解决城镇低收入群体和农村中小学教师住房难问题；实施农村危房改造项目，改善农村住房条件。

　　6．强基惠民工程。筹措资金1.45亿元，实施重点县小型农田水利建设项目、农村居民和学校饮水安全工程；建设通村通屯硬化路36.4公里、安保工程35公里。

　　7．农补惠民工程。筹措资金7700万元，用于农机购置、超级稻种子和农资综合补贴等项目。

　　8．计生惠民工程。筹措资金280万元以上，继续实施免费孕前优生健康检查、建设村级人口计生健康服务室和新家庭文化书屋。

　　9．生态惠民工程。筹措资金7379万元，实施“绿满八桂”造林绿化和森林生态效益补偿项目，建设5000座沼气池，开展5个村环境连片整治。

　　10．土地整治惠民工程。筹措资金1.2亿元，实施10个土地整治和5个地质灾害治理项目。

　　11．粮油惠民工程。筹措资金950万元，建设4家市民放心粮油店，建设粮食应急加工生产线，建立市级粮油质量检测站，设立粮油配送中心。

　　12．警务惠民工程。筹措资金4160万元，建立流动人口服务和管理工作机构，升级改造人口信息管理系统，实施市区视频监控系统三期工程。

　　九、保稳定促和谐

　　促进充分就业。把促进充分就业作为经济社会发展的优先目标，大力推进“乐业”工程，新增就业6万人以上。创新小额信贷运作模式，大力扶持高校毕业生创业。鼓励失地农民、农村转移劳动力参加职业技能培训。积极开发公益性岗位，帮助城镇就业困难人员和零就业家庭实现就业。加强城乡统一的公共就业服务体系和人力资源市场建设。

　　完善社会保障体系。健全各项社会保险制度，扩大社会保障覆盖面，实现基本养老保险、基本医疗保险和新农合制度全覆盖。大力发展社会福利，建设社会福利服务园。加快社会养老服务体系建设，大力发展居家养老服务。加快儿童福利机构建设，建立孤儿保障制度。提高残疾人社会保障和公共服务水平。积极发展慈善事业。

　　强化物价调控。综合运用市场和行政的手段，切实保持物价总水平基本稳定。严格落实“菜篮子”行政首长负责制，稳定和提高蔬菜等农副产品自给能力，保障农副产品市场供应和价格基本稳定。加强对公共产品和公共服务价格的监控。建立社会救助和保障标准与物价上涨挂钩的联动机制，及时向困难群众发放临时价格补贴，保障困难群众基本生活不受影响。

　　创新社会管理。完善社会稳定风险评估工作机制，加大社会矛盾纠纷源头防范力度。加大应急平台建设力度，完善应急处置协调机制。完善人民调解制度，加强道路交通事故、劳资纠纷、医患纠纷等领域行业性、专业性调解组织建设。完善信访工作制度，发挥信访了解民意、服务群众的作用。深入推进平安梧州创建活动，完善社会治安综合治理防控体系，依法防范和严厉打击各类违法犯罪活动，不断提高社会公众安全感和满意度。强化重点行业、重点领域专项整治和安全管理，坚决遏制重特大安全事故发生。强化食品药品安全监管，健全监管机制，提高监管水平，确保食品药品安全。

　　优先发展教育。实施“一镇一公办幼儿园”建设工程。推进义务教育巩固率提高工程、中小学校舍校车安全工程和农村初中改造工程。推进教育布局调整，加快红岭、三龙等新区学校建设，完成梧州高中、梧州一中搬迁。加快市、县职教中心建设，创建国家职业教育教学改革示范试验区。支持梧州学院和梧州职业学院加快发展。实施教师队伍素质提升工程，深化教育改革，提高教育教学质量。

　　发展科技事业。全面实施第五轮科技创新计划，提升科技对经济社会发展的支撑能力。加快科技企业孵化园、科技创业服务中心、科技微型企业青年创业示范园建设。实施专利倍增计划，力争发明专利申请量增长50%以上。加强高新技术产业和战略性新兴产业培育，力争新增高新技术企业2家以上。加快科研学术带头人、科技创新团队和人才小高地建设，提高研发能力和水平。

　　发展医疗卫生事业。积极推进医药卫生体制改革，健全基层医疗卫生机构补偿和绩效考核机制，确保城乡居民每人每年基本卫生服务经费不少于25元，免费提供基本公共卫生服务，实现政府举办的村卫生室国家基本药物制度全覆盖。加快完善城乡卫生服务网络，推进市区8个社区卫生服务中心建设，探索乡村卫生服务一体化管理。加快医疗资源整合，重点推进市人民医院搬迁、工人医院、妇幼保健院等项目建设。支持市红会医院、市中医院创三甲医院，创建“全国中医药工作先进市”。

　　统筹发展其他社会事业。全面推进诚信计生工作，继续稳定低生育水平。以承办广西第十三届运动会为契机，加快市体育中心、体育休闲健身活动场所等项目建设。深入开展国防教育，加强国防动员和后备力量建设。扎实开展双拥共建，巩固和发展军政军民团结。切实做好统计、审计、机构编制、档案、地方志、民族宗教、外事侨务、人民防空、住房公积金、供销合作、国资监管、防震减灾等工作。

　　各位代表！新形势、新任务对政府工作提出了新的更高要求。我们必须牢固树立以人为本、执政为民的理念，切实加强政府自身建设。大力提升机关效能，严格绩效管理、行政问责，认真践行“六戒”，努力建设政治过硬、敢为人先、勇挑重担、朝气蓬勃的公务员队伍，进一步提高执行力、协同力和创新力。严格依照法定权限和程序行使权力、履行职责，自觉接受人大监督和政协民主监督，认真听取各民主党派、工商联、无党派人士和人民团体的意见，高度重视舆论监督和社会公众监督，大力推进政务公开，进一步提高依法行政能力，让行政权力在阳光下运行。切实转变工作作风，把工作的着力点放在基层，把主要精力用在为民办实事、办好事上，做到感情上贴近群众，行动上深入群众，利益上维护群众，真心实意地为群众谋利益，进一步密切同人民群众的血肉联系，使政府工作赢得群众的信任和支持。认真贯彻廉政准则，落实党风廉政建设责任制，完善惩治和预防腐败体系，加强审计监督和行政监察，从源头上防治腐败，努力营造风清气正的社会环境。

各位代表，时代赋予重任，人民寄予厚望。让我们在市委的正确领导下，同心同德，奋发进取，扎实工作，为加快实现富民强梧新跨越而努力奋斗，以优异成绩迎接党的十八大胜利召开！
